# Epigenetics and Methylmercury-Induced Neurotoxicity, Evidence from Experimental Studies

**DOI:** 10.3390/toxics11010072

**Published:** 2023-01-12

**Authors:** Tao Ke, Alexey A. Tinkov, Anatoly V. Skalny, Abel Santamaria, Joao B. T. Rocha, Aaron B. Bowman, Wen Chen, Michael Aschner

**Affiliations:** 1 Department of Molecular Pharmacology, Albert Einstein College of Medicine, Bronx, NY 10461, USA; 2 World-Class Research Center “Digital Biodesign and Personalized Healthcare”, IM Sechenov First Moscow State Medical University (Sechenov University), 119435 Moscow, Russia; 3 Laboratory of Ecobiomonitoring and Quality Control, Yaroslavl State University, 150003 Yaroslavl, Russia; 4 Department of Medical Elementology, RUDN University, 117198 Moscow, Russia; 5 Laboratorio de Aminoácidos Excitadores/Laboratorio de Neurofarmacología Molecular y Nanotecnología, Instituto Nacional de Neurología y Neurocirugía, Mexico City 14269, Mexico; 6 Departamento de Bioquímica e Biologia Molecular, Centro de Ciências Naturais e Exatas, Universidade Federal de Santa Maria, Santa Maria 97105-900, RS, Brazil; 7 School of Health Sciences, Purdue University, West Lafayette, IN 47907, USA; 8 Guangdong Provincial Key Laboratory of Food, Nutrition and Health, Department of Toxicology, School of Public Health, Sun Yat-sen University, Guangzhou 510080, China

**Keywords:** methylmercury, neurotoxicity, transgenerational, histone, DNA methylation, siRNA

## Abstract

MeHg is an environmental neurotoxin that can adversely affect the development of the nervous system. The molecular integrity of chromatin in the nucleus is an important target of MeHg. Low levels of MeHg trigger epigenetic mechanisms that may be involved in long-lasting and transgenerational neurotoxicity after exposure. Emerging evidence has shown that these mechanisms include histone modification, siRNA, and DNA methylation. The MeHg-induced inhibition of neurodifferentiation and neurogenesis are mechanistically associated with epigenetic alterations in critical genes, such as neurotrophin brain-derived neurotrophic factor (BDNF). Further, MeHg exposure has been shown to alter the activity and/or expression of the upstream regulators of chromatin structure, including histone deacetylases (HDACs) and DNA methyltransferase (DNMTs), which may trigger permanent alterations in histone modifications and DNA methylation. MeHg-exposure also alters several species of miRNA that are associated with neurodevelopment. Genetic studies in the *C. elegans* model of MeHg-induced toxicity proposes a potential interplay between exogenous RNAi and antioxidant defense. In this review, we discuss the molecular basis for MeHg exposure-induced alterations in chromatin structure and the roles of histone modifications, siRNA, and DNA methylation in MeHg-induced neurotoxic effects.

## 1. Introduction

Methylmercury (MeHg) is an environmental neurotoxin that may cause long-lasting neurotoxicity in vulnerable populations, such as childbearing women and children [1]. MeHg is a persistent toxicant that is produced from microorganisms in the water system [2]. Environmental exposure to MeHg in the general population comes from eating fish that contain various levels of MeHg (0.02~0.55 ppm; US EPA guideline: 0.30 ppm) [3]. The developing nervous system is a preferential target of MeHg-induced toxicity [4]. The toxic mechanisms of MeHg involves the attachment of MeHg to thiol groups in biomolecules, forming various MeHg-SR complexes [2]. MeHg-SR complexes have lipophilic properties, which tend to partition into cellular lipid regions [5]. Indeed, the increase in partition coefficient after binding (even to cysteine) can explain the LAT1-independent entrance of MeHg in cells [5]. Exposure to this organic mercurial results in the distribution of the metal in the lysosome, mitochondria, and nucleus [6,7,8]. The mechanisms of MeHg-induced cytotoxicity are related to the disruption of homeostasis in a host of cellular physiological functions, including glutathione (GSH) depletion [9], calcium overload [10], loss of mitochondria membrane potential [11,12], and endoplasmic reticulum (ER) stress [13,14]. MeHg can readily cross the nuclear envelope to bind with chromatin components [15,16]. The process may not be involved in the acute toxicity of the metal; however, it could induce changes in the structure of chromatin, leading to long-lasting effects after exposure [17,18,19].

## 2. MeHg, DNA, and Chromatin

**Figure 1:**
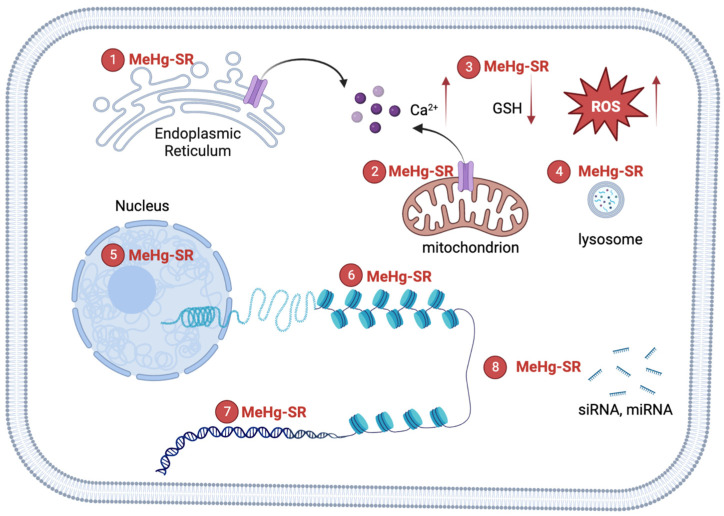
Mechanisms of MeHg-induced neurotoxicity. The formation of MeHg-SR complexes with endogenous thiol-containing biomolecules may increase its lipophilicity, resulting the distribution of the metal into hydrophobic compartments including mitochondria, lysosome, nucleus and other organelles. ① The complexation of MeHg with nascent proteins in the ER may cause ER stress. The black arrow (left): the flow of calcium ions from ER. ② MeHg-SR disrupts mitochondrial respiratory apparatus, leading to the elevation of reactive oxygen species (ROS). Multiples sources of Ca^2+^ contribute to MeHg-induced increase in intracellular Ca^2+^ [10]. The black arrow (right): the flow of calcium ions from mitochondrion. The red arrows (left): the increase of calcium ions; (middle): the decrease of GSH; (right): the increase of ROS. ③ The exchange of MeHg-SR with glutathione (GSH) results in reduction of GSH levels [9]. ④ The majority of MeHg-SR resides in lysosome, which corroborates the structural damage to biomolecules through attachment of the metal to thiol groups [8]. ⑤ MeHg-SR in the nucleus has the potential effects on the integrity of chromatin structures by complexing with histones or DNA [15,33,34]. ⑥ MeHg-RS changes histone post-translational modifications to affect the compactness of chromatin [15]. ⑦ A direct binging MeHg to bases of DNA constitutes the molecular basis for genotoxicity [20]. ⑧ MeHg-SR can interrupt biogenesis of siRNA and/or miRNA, leading to alterations in siRNA and/or miRNA-mediated gene regulations [40].

MeHg has a characteristic binding activity to the C- or N-containing moiety groups in the bases of DNA [20]. The physicochemical property of MeHg makes it very useful in the study of DNA structure. One of its compounds, methylmercury hydroxylate (MeHgOH), was used as a chemical probe to investigate DNA secondary structure and unpaired bases [21]. As it readily reacts with the purine and pyrimidine residues of nucleic acids, MeHgOH was also used as a reversible denaturing agent for DNA agarose gel electrophoresis [22]. The binding of MeHgOH to different bases in singular and duplex DNA was utilized for the detection and quantification of single-stranded DNA in duplex DNA samples [16]. These in vitro binding studies, which employed MeHgOH as the chemical species of MeHg, are in line with the genotoxic effects of MeHg [23,24,25,26,27]. The binding property to DNA can be changed with different ligands complexed to MeHg: for example, methylmercury chloride (MeHgCl), another widely used experimental chemical species. MeHgCl can interact with cysteine to form a major bioavailable form, MeHg-S-Cys [28]. The exchange of dissociable anions in MeHg complexes with thiol groups in other biomolecules dictates its molecular toxicity [2,29]. A cell culture study has shown that both MeHgOH and MeHgCl can cause cytotoxicity and genotoxicity, and MeHgCl is more toxic to SH-SY5Y cells than MeHgOH [30]. The differential toxic effects between MeHgOH and MeHgCl can be attributed to the stronger lipid partition coefficient of MeHgCl [5]; however, in a complex system in the cell, the genotoxic effects are probably mediated by their interaction with thiol-containing critical chromatin proteins (Figure 1) [2]. The integrity of DNA replication may be compromised after MeHg exposure, as corroborated in many studies on MeHg-induced genotoxicity [23,24,25,26,27]. These studies pave the way for the understanding of MeHg-induced genotoxic effects and its potential impact on the structure of nucleosomes and chromatin.

Although MeHg has a high affinity for sulfhydryl groups [31], its binding partners in the cellular system are also governed by a number of factors, such as exchange reactions and protein-specific structural and thermodynamical factors [2]. MeHg can bind with a variety of biomolecules in the nucleus, including DNA, histones, and non-histone protein components [15,32,33]. Likewise, many factors influence the propensity of the binding of MeHg to DNA bases, including the concentration of MeHg, temperature, base composition, ionic strength, and pH, to name a few [20]. A study in HeLa S3 suspension-culture cells has shown that the binding of MeHgCl to DNA and chromatin is a rapid process and could readily take place after the exposure [33]. A primary cell culture study in mouse fetal astrocyte has shown that MeHgCl exposure can competitively block the histone binding sites of the histone specific dye, N-(3-pyrene)maleimide, which specifically labels the cysteine groups in histone H3 of nucleosomes [15]. The blockage of the dye binding was gradually increased upon prolonged exposures in the in vitro model [15]. MeHg exposure also disrupts the complexing of histones with DNA. An in vitro study has shown that MeHgOH (1~10 μM) interferes with the binding of DNA by histones H3 and H4 [34]. A higher concentration of MeHgOH (10~32 μM) disrupted the complexing of DNA with the histones H2A and H2B [34]. The structure of chromatin plays an important role in gene regulation, which is predominantly regulated by histone proteins [35]. The post-translational modifications of histones, such as methylation and acetylation, regulates chromatin compactness, transcriptional activity, and genome functions [36]. Further, the regulation of gene expression at the transcriptional level involves epigenetic programs that are encoded by histone post-translational modifications, which determine the compactness of chromatin and transcriptional activity [37]. For example, increases in histone H3K4 methylation around the transcriptional start site (TSS) are associated with active transcription while high levels of H3K9 methylation at this region are associated with gene repression [38]. The regulatory mechanism during neuronal development and differentiation spatially and temporally invokes the modification of histones to fulfill gene regulation purposes [39]. Thus, the potential effects of MeHg on the post-translational modification of histones likely involves a direct binding of MeHg to the components of chromatin (particularly to thiol-containing proteins), leading to interference in chromatin compactness and structure. Though MeHg can interact with nucleophilic N-atoms found in nitrogenous bases forming the DNA nucleotides, the thiol-containing proteins found in the chromatin are possibly MeHg’s preferential targets. However, our knowledge on how MeHg disrupts the physiology and biochemistry of chromatin is still elusive. Recent experimental studies have shown that low levels of MeHg (nM) induce changes in histone post-translational modifications and DNA methylation, both of which may serve an important base for the long-lasting and transgenerational effects after the exposure [17,18].

## 3. MeHg and Neurogenesis

Developmental MeHg exposures not only retard tissue development [41,42], but they also trigger long-lasting effects secondary to altered neuronal differentiation and neurogenesis [19,43]. Studies in in vitro cell cultures, including primary cell culture, immortalized neuronal cell lines, and human iPSC derived neurons, as well as animal models, suggest that exposure to low-level MeHg at the early developmental stage or parental lines induced long-last effects at the mature stage or even in future generations [17,18,19,44]. These effects are hardly explained by the persistence of MeHg in cells, as the mercury level in offspring returned to control levels [17,19]. For example, in a differentiating neurocortical human-induced pluripotent stem cell (hiPSC) model, a persistent change in gene expression related to a large number of biological processes was evident weeks after the removal of MeHgCl from the culture media [43]. In the same system, an inhibition or delay of neuronal differentiation was also noted in hiPSC differentiating into cortical glutamatergic (GLUergic) neurons without evidence of changes in neuronal fate [45].

Neurotrophin brain-derived neurotrophic factor (BDNF) has important functions in neuronal differentiation, maintenance, and neurogenesis [46]. Male mice with perinatal MeHgOH exposure (0.5 mg/kg/day for 2 weeks) exhibited depression-like behaviors in the forced swim test at the age of 9 weeks [18]. These mice had a significantly longer immobility time than the controls during the forced swim test, and the effect could be abolished by the treatment with the selective serotonin reuptake inhibitor fluoxetine (0.08 mg/mL) for 3 weeks. These observations were associated with the downregulation of BDNF mRNA levels in the dentate gyrus (DG) region of the hippocampal formation revealed by fluorescence in situ hybridization (FISH). Additionally, the chromatin of the promoter IV of BDNF showed a long-lasting increase in histone H3K27 tri-methylation and a decrease in histone H3 acetylation in mice aged between 12 and 14 weeks following perinatal MeHgOH exposure. The decrease in histone H3 acetylation was mechanistically associated with the MeHg-induced down-regulation of BDNF mRNA and depression-like behaviors. The DG region is one of the major brain areas with active neurogenesis at the adult stage [47]. The evoked expression of BDNF is required for hippocampal neurogenesis in rodents in response to environmental enrichment [48]. Another study in mice showed that environmental enrichment triggered significant changes in methylation levels of histone H3 in the BDNF promoter regions, which is associated with the increased expression of BDNF mRNA in the hippocampus and the neurodifferentiation of granule cells in the DG region [49]. The study supports the notion that the epigenetic mechanisms of histone post-translational modifications are highly involved in the environmental factors-induced upregulation of BDNF and neurogenesis.

Environmental chemical exposures have been shown to trigger epigenetic mechanisms that alter the stress response pathway in the brain including, the hypothalamic-pituitary-adrenal (HPA) axis, increasing disease vulnerability [50]; however, in the rat model with perinatal exposure to a mixture of chemicals, including MeHgCl, the exposure failed to alter the DNA methylation level in the promoter region of the glucocorticoid receptor (GR) gene [51]. Although the study mimicked real-life exposure to a chemical mixture, it is difficult to interpret the individual effect of MeHgCl. Nevertheless, the involvement of epigenetic programs in response to MeHg exposure was demonstrated in controlled experimental settings [18,52,53].

In SH-SY5Y cells, MeHgCl exposure (1 μM, 24 h) reduced the level of histone H4 acetylation. The pan-histone histone deacetylases (HDAC) inhibitor trichostatin A (10~50 nM) dose-dependently reduced the MeHgCl-induced cell death and repression of histone H4 acetylation [53]. In the cerebellum of MeHgCl-exposed mice (subcutaneous injection, 10 mg/kg, 10 days), the level of histone H4 acetylation was downregulated up to more than 50%; however, the amount of histone H4 acetylation in the cortex was well preserved [52]. HDACs regulate histone H4 acetylation. Of the six isoforms of HDACs (1~6), isoform 4 expression was significantly increased by MeHgCl exposure (1 μM, 24 h). Chromatin immuno-precipitation (ChIP) analysis showed that the induction of HDAC4 was regulated by the increased binding of the transcription factors specificity proteins1 (Sp1) and Sp4 to the promoter region of the HDAC4 gene, which appeared to be triggered by p38 signaling. Consequently, the binding of HDAC4 to the BNDF IV promoter was increased, leading to the reduction of BNDF expression in SH-SY5Y cells following MeHgCl exposure (1 μM, 24 h). The study also showed that MeHgCl-induced reduction in BNDF expression and the upstream pathway of p38/HDAC4 were mechanistically linked to the cell death triggered by the exposure [53].

## 4. MeHg, miRNA, and RNA Interference (RNAi)

MeHg-induced neurotoxicity and repression of BDNF likely activates a pathway downstream of microRNA (miRNA), which are single stranded, non-coding RNA molecules (21–23 nucleotides), and involved in RNA silencing and transcriptional regulation of gene expression [54]. In rat embryonic cortical neurons, MeHg exposure (1 μM, 24 h) reduced the expression of miR-206 and BDNF [55]. The overexpression of miR-206 reverted MeHg exposure-induced BDNF reduction via modulation of repressor element-1 silencing transcription factor (REST) and specificity protein 4 (Sp4). Specifically, the overexpression of miR-206 promoted the binding of the transcription factor JunD to the promoters of repressor element-1 silencing transcription factor (REST) and specificity protein 4 (Sp4), leading to an upregulation in the expression of BNDF [55]. The importance of miRNA in MeHg-induced neurotoxcity was also demonstrated in a miRNA profiling study in a human pluripotent cell model, in which MeHgCl exposure induced the alteration in the expression of miRNAs invovled in neural development and the ubiquitin-proteasome pathway [56].

A number of miRNAs were also highlighted in the invertebrate model of MeHg-induced toxicity [40]. In the *C. elegans* model of MeHgCl exposure, synchronized eggs were cultured in plates supplemented with 10 μM MeHgCl for 48~56 h. Following the exposure, miRNA-seq analysis showed that miR-37-3p, miR-75-3p, miR-70-3p, and miR-41-5p were decreased 5.7~7.8 fold in MeHgCl exposed worms [40]. The majority of *C. elegans* individual miRNA is not essential for development or viability [57]; however, they have important regulatory roles in embryonic morphogenesis [58], developmental timing [59], and numerous physiologic processes including locomotion, egg laying, and dauer entry [60]. The significance of these miRNAs in MeHg-induced neurotoxicity remains elusive [40]. Intriguingly, miRNAs also regulate RNAi sensitivity to endogenous and exogenous siRNAs [61]. Several studies have shown that enzymes in the siRNA biogenesis might be involved in MeHg-induced toxicity in *C. elegans* [62,63,64].

In *C. elegans*, RRF-3 is an RNA-dependent RNA polymerase (RdRP) which is required for primary 26G RNAs biogenesis in germline and early larval development [65]. RRF-3 generates endogenous primary short interfering RNAs (siRNAs) to repress gene expressions in spermatogenesis [66], for the clearance of maternal transcripts in zygotic development [67]. The genesis of abundant secondary 22G RNAs is dependent on another RdRP, RRF-1 [68,69]. In the *C. elegans* model of MeHgCl-induced toxicity [63], it was shown that a strain harboring the *rrf-3* gene with a partial deletion of the coding region was resistant to MeHgCl-induced toxicity. The mutant strain (*rrf-3*(pk1426)) is hypersensitive to RNAi and resistant to MeHgCl-induced toxicity [62,63]. Another independent study showed that *C. elegans* mutant strains, including (*rrf-1*(pk1417)) and (*rrf-3*(pk1426)), were also resistant to MeHgCl-induced toxicity [64]. Additionally, the molecular components in the early steps of siRNA genesis may also be involved in MeHg-induced toxicity. *Pash-1* encodes the *C. elegans* ortholog of DGCR8/Pasha, which is required in the first step for the cleavage of the primary transcript and miRNA processing in the nucleus [70]. The gene of nuclear RNAi defective-2 (*nrde-2*) encodes one of *C. elegans* homologs of Argonaute, which is required for siRNA-mediated transcriptional silencing in nuclei (nuclear RNAi) [71,72], and heterochromatin assembly by siRNA [73]. The study on genetic mutant strains of *pash-1* and *nrde-2* showed that these worms were hypersensitive to MeHgCl-induced toxicity, suggesting that the endogenous RNAi pathway might be evoked against toxic effects of MeHgCl [64].

The association between RNAi and MeHg-induced toxicity seems hard to be explained by MeHg’s well-established mechanisms, namely oxidative stress, mitochondria toxicity, ER stress, calcium dysregulation, or genotoxicity [9,10,11,12,15,16,23,24,25,26,27,74]. A recent study in the *C. elegans* model showed that a worm strain with a null allele of *rrf-3* had elevated levels of gene expression in the detoxification pathway [63]. As one of the mechanisms for gene regulation, RNAi is also a host defense mechanism against exogenous genetic elements, such as viral infection [65,75]. The worm harboring the *rrf-3* null mutation is hypersensitive to exogenous RNAi, which is attributed to the downregulation of endogenous RNAi and the competition between exogenous and endogenous RNAi for the common RNAi machineries [76,77,78]. The RNAi pathway mediated by siRNA from a viral genome is important in facilitating antiviral immunity during viral infection [79]. Meanwhile, the interaction between viral proteins and components of mitochondrial respiratory apparatus promotes ROS production [80]. Therefore, the increase in the detoxification pathway in the *rrf-3* mutant strain is likely coupled to increased sensitivity to exogenous RNAi, both of which can be mobilized in the surveillance of and against the virus’ infection [80,81]. This may constitute the basis for the high antioxidant potential in the *rrf-3* mutant worms against MeHg-induced oxidative stress.

## 5. Transgenerational Neurotoxicity of MeHg

The two important targets of MeHg-induced toxicity, including cell differentiation and epigenetics of germline cells, may serve the bases for understanding the long-term as well as transgenerational neurotoxicity of early MeHg exposures at low levels [43,45,82].

ChIP sequencing (ChIP-seq) can reveal changes in histone modifications across the genome level. Such a study showed that many gene loci can be epigenetically modified by MeHg exposure [83]. In the *C. elegans* model, synchronized larvae stage 1 (L1) worms were exposed to MeHgCl (10 μM) until the L4 stage. ChIP-seq data showed that the levels of H3K4me3 were significantly increased in genes involved in cellular detoxification, mitochondrial protein quality control, unfolded protein response, and cuticle formation and maintenance [83]. The increased levels of H3K4me3 reveals the active transcription of associated genes. In L4 stage worms developed from eggs exposed to MeHgCl in utero, the levels of H3K4me3 were also significantly increased in loci that are involved in the detoxification of xenobiotics, such as the glutathione S-transferases (GSTs) [83]; however, the expression of the genes was not increased, which suggests that other inhibitory mechanisms might collectively control the gene expression in the context of H3K4me3 induction. Albeit there was a long-lasting change in H3K4me3 following in utero MeHgCl exposure, acute MeHgCl exposure increased the expression of GSTs in worms with or without in utero MeHgCl exposure.

DNA methylation in the germline is also affected by MeHg exposures. In the zebrafish model, the animals were exposed to MeHgCl (30 nM, 24 h) at the embryonic stage. In the F2 generation that had comparable mercury levels to the background level in controls (5 ppb), the fish showed visual deficits and hyperactivity, affirming a transgenerational neurotoxicity of ancestral MeHgCl exposure [17]. To exclude bias from DNA methylation analyses in samples collected from multiple tissues, the study specifically focused on sperm DNA methylation. The sperm DNA epimutation is a heritable alteration in DNA methylation that can be passed down to future generations and is one of the molecular mechanisms for transgenerational effects [84]. The analysis of sperm DNA methylation in the F2 showed that there were differential DNA methylation regions (DMRs) in the line from ancestral MeHgCl exposure [17]. Intriguingly, the numbers of DMRs mapped to all chromosomes in the F2 generation was higher than the F0 generation. Specifically, the densest DMRs cluster was distributed on the chromosome 4 in the F0, whereas the DMRs clusters in F2 were less localized but heavily distributed among chromosomes. It appeared that the DMRs clusters frequently occurred in the genomic region of CpG deserts in both F0 and F2. The majority of DMRs was unique for the F0 and F2 generation. In the F2 generation, the DMR associated genes included those involved in the process of signaling, metabolism, receptors, and proteases [17]. Despite the strong effect of MeHg on sperm epimutation, the global DNA methylation levels were not significantly changed in F1 and F2 embryos of female fish exposed to MeHg-cys (10 mg/kg, 47 days) in another Zebrafish model [82]. As the oocytes of mother fish were exposed to MeHg-cys, the F1 and its embryo might still have some level of mercury, which compounds the possible role of transgenerational mechanisms in DNA methylation in the study [82].

The inheritance of epigenetic programs in heterochromatin is dependent on sequence-specific elements (*cis* elements), including DNA silencers and noncoding RNAs to recruit additional histone modification factors to reestablish and inherit epigenetic state [85]. The inherited parental histones can be recognized by these cis elements by recruiting multiple protein complexes and working cooperatively with histone modifications to spread and reestablish histone landmarks in heterochromatin [85]. Similarly, parental DNA methylation state can be established by recruiting DNA methyltransferase (DNMTs) to hemimethylated daughter DNA to reestablish parental DNA methylation patterns [86].

The transgenerational effects of MeHgOH were demonstrated in a study in an in vitro model of rat neural stem cells (NSCs) [19]. NSCs showed cell cycle arrest after exposure to 5~10 nM MeHgOH for 48 h. The effect on the cell growth was persistent in the daughter cells after the second passage. The daughter cells had a minimal level of mercury, which is comparable to the background mercury level. This observation is consistent with cell cycle arrest and an inherited effect of parental MeHgOH exposure. In addition, the global methylated DNA (5-methylcytosine) level in the genome was decreased in both parental and daughter cells along with the persistent induction of genes (p16 and p21) involved in cell cycle regulation [19]. DNA methylation levels are regulated by DNMTs [87]. The study also showed that there was an inherited effect on the expression of DNMT3b, an enzyme for *de novo* DNA methylation; however, the expression of DNMT1 was not affected [19]. The induction of cell cycle regulators (p16, p21, and p53) by low level MeHgCl (10 nM for 24 h) was further demonstrated in a model of immortalized human neural progenitor cells (ihNPCs) [88]. In the same model, the expression of miR-30d and miR-25 was reduced following MeHgCl exposure. The overexpression of miR-25 partially reverted the MeHgCl-induced overexpression of p53 and genes related to mitochondrial biogenesis.

The repression of DNMTs might be involved in MeHgCl-induced developmental neurotoxicity. In an experimental study on captive mink exposed to MeHgCl, brain DNMT activity was decreased more than 50% in the animals with brain mercury greater than 1 ppm [89]. There was a slight inverse association between brain mercury and DNA methylation in the occipital cortex of the mink [89]; however, the injection of MeHgCl into chicken embryos or dietary MeHgCl exposure in female yellow perch failed to induce analogous effects, which raises the possibility that the changes in DNA methylation and DNMT activity following MeHg exposures are species-specific or treatment route specific [89]. The repression of DNA methylation and DNMTs may be specific to mammalians exposed to MeHg in the natural environment. In an observational study in male polar bears, brain mercury levels (0.03~0.18 ppm) were inversely associated with genomic DNA methylation in the lower brain stem region [90].

Additional observations in lab animals supported the notion that the effect of MeHg exposure on DNMT expression was isoform specific. Embryonic MeHgCl exposure (3 mg/kg, 3 days) decreased neurite length in the mouse fetal cerebral cortex at embryonic day 19 [91]. In the fetal cerebral cortex, methylated DNA (5-methylcytosine) was significantly increased. Meanwhile, there was a greater than three-fold increase in the expression of DNA methyltransferase 1 (DNMT1); however, the expression of DNMT3A or DNMT3B was unaffected. Both the acetylation of histone 3 (AcH3) and histone H3 at lysine residue 14 (AcH3K14) was decreased. The increased expression of DNMT1 may represent a feedback response to the decreased activity of the enzyme, which needs further evidence. In addition, the level of HDAC3 and HDAC6 was also increased [91]. In the same study in differentiated Lund Human Mesencephalic (LUHMES) cells that originate from the human mid-brain, MeHgCl exposure (1 nM, 6 days) inhibited the neurite outgrowth. The increase in methylated DNA (5-methylcytosine) was also noted in the in vitro model. Interestingly, all three isoforms of DNMTs (DNMT1, DNMT3A, and DNMT3B) were significantly increased in the model. In addition to the decrease of AcH3 and AcH3K14, the trimethylation of histone H3 at lysine 27 (H3K27me3) was increased more than three-fold. Moreover, a nonselective DNMT inhibitor 5-Azacytidine (1 μM) abrogated the inhibitory effect of MeHgCl exposure on neurite outgrowth. Similar effects were observed in cells treated with HDAC inhibitors, suggesting the involvement of epigenetic mechanism in MeHgCl-induced developmental neurotoxicity [91].

## 6. Conclusions

**Figure 2:**
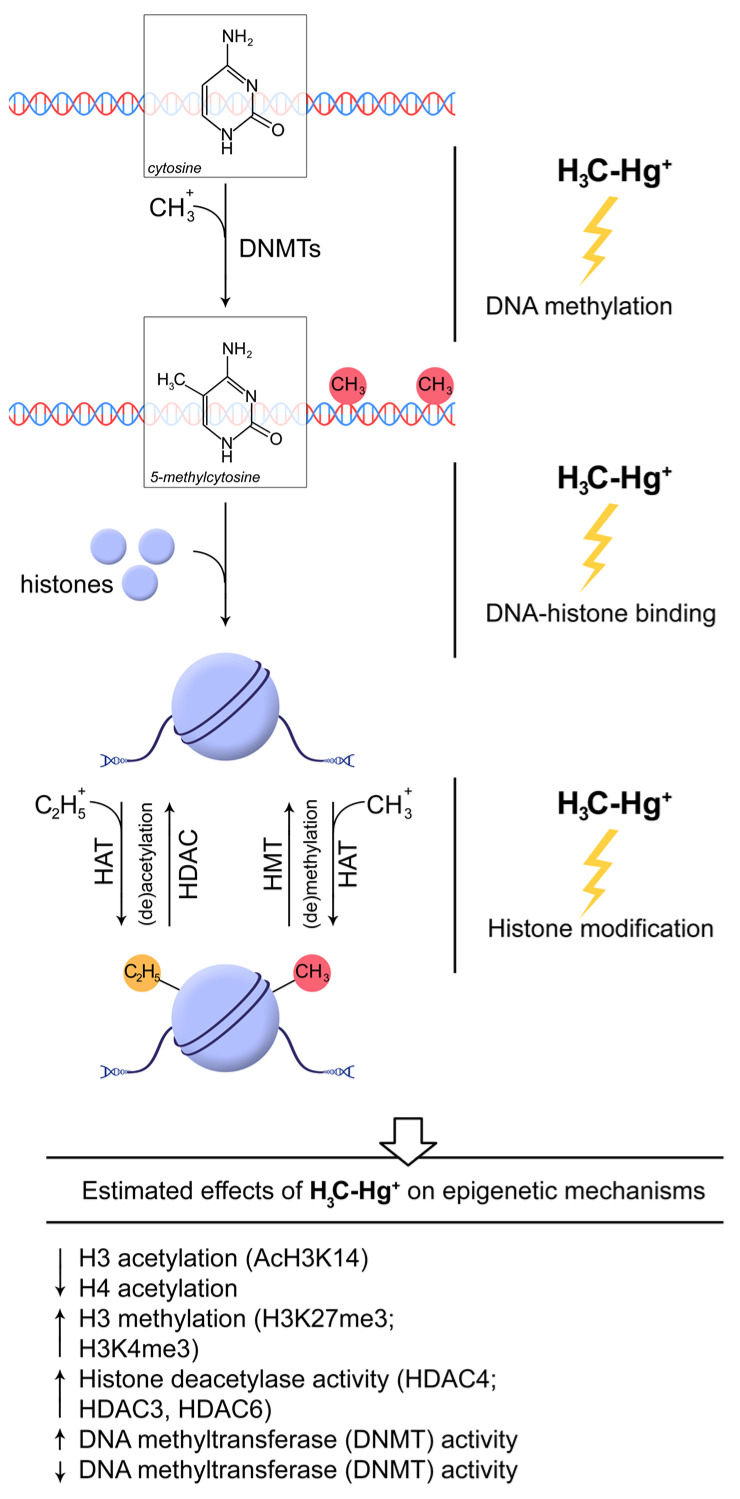
Epigenetic mechanisms potentially involved in MeHg-induced neurotoxicity [18,52,53,83,89,90,91]. Epigenetic effects of MeHg exposure may be mediated through a number of mechanisms including modulation of DNA methylation, histone-DNA binding, and histone modifications. Specifically, MeHg was shown to modulate DNMT activity resulting in significant changes in DNA methylation, although both up-regulation and down-regulation of DNMT was reported. Alteration in DNA-histone binding may ultimately result in impaired nucleosome formation, also corresponding to the earlier reported effect of MeHg on chromatin compactness and structure. MeHg was also shown to affect histone modification with reduction of histone 3 and 4 acetylation due to up-regulation of HDAC4, as well as HDAC 3 and 6 activity. Although stimulatory effect of MeHg on histone 3 methylation was demonstrated, the particular impact of MeHg on regulatory enzymes including HMT and HDM is still unclear. It is expected that these mechanisms may at least partially mediate the impact of MeHg on molecular targets underlying its neurotoxicity (e.g., inhibition of BDNF signaling).

Multiple mechanisms for cellular surveillance and homeostasis are involved in MeHg exposure-induced neurotoxicity, and these mechanisms are highly related to the physiochemical properties of the metal. The chromatin structure is a direct target of MeHg exposure. Additionally, MeHg exposure can induce long-lasting or even transgenerational alterations in histone modifications and DNA methylation (Figure 2). Recent advances in epigenetic mechanisms of neurotoxicity have broadened our views on this metal. MeHg exposures have been shown to alter the activity and/or expression of enzymes involved in histone modifications and DNA methylation, such as DNMTs and HDACs. The intriguing interplay between exogenous RNAi and oxidative stress deserves further investigations. Although these epigenetic machineries are highly involved in MeHg-induced neurotoxicity, more studies are needed to establish a molecular pathway that can serve as the basis for MeHg exposure-induced epigenetic effects in different animal models.

## Data Availability

No new data were created or analyzed in this study. Data sharing is not applicable to this article.
